# Validation of the World Health Organization Well-Being Index (WHO-5) among medical educators in Hong Kong: a confirmatory factor analysis

**DOI:** 10.1080/10872981.2022.2044635

**Published:** 2022-03-11

**Authors:** Linda Chan, Rebecca K. W. Liu, Tai Pong Lam, Julie Y. Chen, George L. Tipoe, Fraide A. Ganotice

**Affiliations:** a Department of Family Medicine and Primary Care, The University of Hong Kong, Pokfulam, Hong Kong, China; b The Bau Institute of Medical and Health Sciences Education, The University of Hong Kong, Pokfulam, Hong Kong, China; c Department of Family Medicine and Primary Care, The University of Hong Kong - Shenzhen Hospital, Shenzhen, Guangdong, China

**Keywords:** Well-being, WHO-5, medical educators, psychometrics, confirmatory factor analysis

## Abstract

**Background:**

The COVID-19 pandemic has exacerbated the pre-existing global crisis of physician burnout. Physician and particularly medical educator well-being, has come into focus as educators can influence their own and learners’ well-being. Measuring this construct is one important step towards promoting well-being in the work and learning environments. The 5-item World Health Organization Well-Being Index (WHO-5) has been validated in different populations worldwide for assessing well-being. Yet, its psychometric acceptability remains unexplored among medical educators in Asia including Hong Kong (HK). This study evaluates the validity of the WHO-5 when used among HK medical educators.

**Method:**

Using data from 435 medical educators, we employed combined within-network (confirmatory factor analysis; CFA) and between-network approaches (correlation and regression) to scale validation.

**Results:**

CFA results indicated that our data fit the a priori WHO-5 model, suggesting structural validity. Results of comparison of means indicated no gender differences, but there were significant differences when participants were compared by age and professional backgrounds. Resilience predicted well-being as measured by the WHO-5, suggesting construct criterion validity.

**Conclusions:**

Our findings extend the validity evidence for the WHO-5 to HK medical educators examined in this study. This enables their well-being to be assessed when evaluating the impact of future well-being programmes.

## Introduction

Globally, physician well-being has garnered increasing research attention in recent years. This reflects the alarming rates of psychological distress [1,2], suicidal ideation [3], and suicide among physicians [4], in addition to the associated negative impact on patient care and healthcare systems [5]. Subjective well-being is a broad concept consisting of positive affect, life satisfaction, fulfilment and positive functioning, as well as the absence of negative emotions [6]. The importance of promoting well-being among healthcare professionals has been amplified by the current Coronavirus Disease 2019 (COVID-19) pandemic with the World Health Organization (WHO) highlighting concerns about the potential negative ramifications of this unprecedented crisis on the psychological well-being of healthcare providers [7]. A meta-analysis conducted during the COVID-19 outbreak reported that among 79,437 healthcare workers across 24 countries, the pooled prevalence of psychological distress was 46.1%, stress 40.3%, burnout 37.4%, anxiety 34.4%, depression 31.8%, insomnia 27.8% and post-traumatic stress syndrome 11.4% [8]. Consequently, the academe is called to respond by identifying strategies to enhance well-being among healthcare professionals.

Medical educators in particular could have their well-being compromised by extra teaching obligations on top of their clinical role. For example, 69.6% of otolaryngologist-educators demonstrated burnout symptoms, which was higher than those in practice [9]. Such results are concerning, since reduced well-being in educators could adversely impact learners’ well-being, for example through suboptimal behaviours and role-modelling [10]. In this context, the assessment of medical educator well-being, its antecedents and consequences become even more crucial.

### World Health Organization Well-Being Index

The 5-item World Health Organization Well-being Index (WHO-5) is a widely used instrument designed to measure subjective well-being [11]. It is brief, taking less than a minute to complete and is easy to score. This makes it a time efficient and efficacious tool to monitor well-being. Currently, the WHO-5 has been translated into over 30 languages including Chinese, French, Russian and Arabic [12]. The instrument has been used in research projects globally [11], validated in various populations, and used for investigating the relationship of well-being with other constructs such as resilience, depression and suicidal ideation [13–15].

Within the wider literature, there has been limited prior research examining how well-being varies among the non-patient population according to different variables, for example age, gender and professional background. With regards to age, inconsistencies have been reported with older age being associated with poorer well-being in German emergency medical service professionals [16]. In contrast, older Danish general practitioners had higher well-being compared to their younger counterparts [17]. However, age was not observed to have an impact on well-being among frontline physicians and nurses in Oman during the COVID-19 pandemic [18].

With regards to gender, inconsistencies also exist with no gender differences observed among Canadian resident physicians [19], U.S. medical students, interview candidates for residency, and internal medicine residents plus faculty [20], as well as Dutch emergency department staff during the first COVID-19 wave [21]. In contrast, well-being was noted to be lower among female paramedics and emergency physicians in Germany [16]. Interestingly, female office workers in Italy [22] and South Korea [23] also had lower subjective well-being on the WHO-5 than their male colleagues.

There is a dearth of research exploring differences in well-being across various professional backgrounds. One such study among Dutch emergency department doctors, nurses, nursing assistants and administrators found that doctors had a significantly higher mean WHO-5 score compared to other staff during the peak of the first COVID-19 wave [21].

With cross-country comparisons on well-being being undertaken [24], meaningful comparison can only take place when validity evidence has been found to support the use of an instrument in a new group. For this purpose, we examined the psychometric applicability of the WHO-5 among a sample of Hong Kong (HK) medical educators with the end of promoting a conversation that will help to enhance their well-being in the workplace. Furthermore, given the limited research discussed earlier, this study seeks to understand whether the WHO-5 is able to differentiate between the well-being of medical educators when grouped according to age, gender and professional background. To our knowledge, no empirical studies related to the psychometric acceptability of the WHO-5 among HK medical educators have been done. Addressing this knowledge gap provides us opportunities to understand the well-being of medical educators better, which has implications since they can also influence the well-being of their learners through the formal, informal, and hidden curricula [25].

### Aim

Utilizing the strengths of both within-network and between-network approaches to construct validity, this study aims to gather validity evidence of the WHO-5 among a sample of HK medical educators. In particular, we aim to: (1) examine if the data from a sample of this population fit the a priori WHO-5 model through confirmatory factor analysis (CFA); (2) determine if the scale is able to differentiate between the well-being of HK medical educators when grouped according to age, gender, and professional background; (3) examine the correlation of well-being as measured by the WHO-5 with other theoretically relevant constructs such as resilience.

## Methods

### Approaches to scale validation

Construct validation is one of the popular approaches to scale validation [26,27]. This can be conducted through within-network or between-network studies. Within-network validation is an internal construct validation technique which can be accomplished through reliability and factor analysis. Whereas, between-network validation is an external construct validation method performed by examining patterns of relationships between the scale and other theoretically related variables. This dual approach to construct validation is said to be robust and has been used in validation studies [27].

Applying these approaches to the current research, we conducted a within-network study by examining the factor structure through CFA to clarify if our data involving HK medical educators fit the a priori model. We assumed that all five items in the WHO-5 would load into a single factor as defined by the a priori model. We also examined the internal consistency reliability and interfactorial correlation. To establish between-network construct validity, we examined the relationship between well-being and resilience. We hypothesised that resilience predicts well-being in medical educators. This is consistent with previous studies indicating the positive relationship between resilience and well-being [15,28,29].

### Participants, sample size calculation and procedures

The research participants were the academic, academic-related staff and honorary teachers with the LKS Faculty of Medicine at the University of Hong Kong (HKU). These educators had multidisciplinary backgrounds, and were involved across the entire medical education spectrum, in addition to administration and research. HKU’s Human Research Ethics Committee approved this study (reference no.: EA200136).

For calculating the minimal sample size necessary, a previous study showed a medium correlation (*r* = 0.378) between resilience and well-being in HK nursing students [15]. Assuming a conservative estimation for the small correlation (*r* = 0.2) between resilience and well-being, a minimum sample size of 319 participants was required to detect the difference at 95% power with a significance level of 0.5. This was also consistent with literature recommending a sample size greater than 200 when conducting CFA [30,31].

All HKU medical educators based in HK were eligible to participate if they were involved in teaching medical students, trainees, and/or doctors. They were recruited using emails that contained links to an online questionnaire. A participant information sheet was accessible from the questionnaire’s front page. Electronic informed consent for voluntary participation was obtained [32] and convenience sampling was used.

Since January 2020, the COVID-19 outbreak in HK resulted in an emergency pivot from conventional face-to-face to virtual teaching. This caught many medical educators off guard and may have potentially challenged their well-being. Data collection for this study started in October 2020 and continued until January 2021 during HK’s fourth COVID-19 wave. The timing of the data collection was critical as it could have captured the well-being issues related to the impact of this sudden transition to online teaching and learning.

### Questionnaire design

An anonymous, self-administered online questionnaire was developed using Qualtrics (https://www.qualtrics.com/). It incorporated sociodemographic questions and the following validated instruments.

#### World Health Organization Well-Being Index (WHO-5)

This short, self-administered measure assesses subjective well-being over the last two weeks. Its five positively worded items are rated on a Likert scale ranging from 0 (at no time) to 5 (all of the time). The total raw score ranges from 0 to 25, which is then multiplied by 4 to convert it into a percentage (0 to 100). Increasing scores represent greater well-being. A score of 50 or below represents poor well-being and is used to screen for depression. A systematic review demonstrated that the WHO-5 had high clinimetric validity and broad applicability across study fields [11].

#### Connor-Davidson Resilience Scale

The validated 2-item Connor-Davidson Resilience Scale (CD-RISC2) was included for self-assessing resilience [33]. Participants rate the items using a 5-point Likert-scale ranging from ‘not true at all’ (0) to ‘true nearly all of the time’ (4). Total scores range from 0 to 8, with higher scores reflecting greater resilience. Its test-retest reliability, convergent, and divergent validity have been established [33]. Furthermore, the CD-RISC2 was found to be reliable and valid using a large random sample drawn from HK’s general population, as well as highly correlated with the original 25-item CD-RISC [34].

Feedback was obtained from expert medical educators and pilot testing of the questionnaire involving a sample of educators was conducted before it was finalised and distributed among HKU’s medical educators [35]. The questionnaire took about 5–10 minutes to complete and three electronic reminders were distributed over three months. As tokens of appreciation, coffee e-vouchers were sent to the first 150 participants who provided their emails.

### Data analysis

CFA is a popular form of psychometric assessment appropriate when a researcher has some previous knowledge of the underlying latent variable structure of the a priori model. This approach examines the extent to which a highly constrained a priori factor structure is consistent with the sample data [36]. In contrast to exploratory factor analysis, CFA works deductively in validating the factor structure of the a priori model [37].

To establish both within- and between-network construct validity, we first examined descriptive statistics and normality of the data. Second, we performed CFA using the maximum likelihood estimation approach to assess whether the hypothesised unidimensional WHO-5 measurement model was applicable to the participants. To understand the model fit, we used a number of goodness-of-fit indices: goodness-of-fit index (GFI), Tucker–Lewis Index (TLI), comparative fit index (CFI), root mean square error of approximation (RMSEA), standardised root mean square residual (SRMR), Chi square and Chi square to degrees of freedom ratio. Values of above 0.90 for GFI, TLI, and CFI are deemed acceptable, while RMSEA and SRMR should be below 0.08 [38]. The Chi square should also be non-significant. However, researchers have found that this is usually overly sensitive to sample size differences [39].

We used one-way analysis of variance to examine age group differences in well-being, and independent t-tests to examine differences in well-being by gender, as well as professional background. To establish the between-network construct validity of the WHO-5, we used linear regression analysis which determines the relationship between a single predictor variable and an output variable. In this study, we used resilience as measured by the CD-RISC2, as a predictor of well-being as assessed by the WHO-5. All analyses were conducted using Statistical Package for Social Sciences (SPSS Version 26) and Analysis of a Moment Structures (AMOS Version 26) statistical software (IBM Corp., Armonk, NY, USA).

## Results

### Participants

There were 435 medical educators participating from 1,902 who received an invitation, giving a response rate of 23%. As summarised in Appendix Table 1, the majority of them were male (66.7%), aged 40 to 49 years old (34.3%) and ethnically Chinese (93.8%). Professionally, most were in full-time employment (60.7%), had a specialist qualification (44.3%) in terms of the highest professional degree obtained, and had been working as a health professions educator for 20 or more years (42.1%). All participants had roles in teaching and either clinical or science backgrounds. Among the clinical practitioners, the majority were in medicine (79.9%). Those with science backgrounds did not provide clinical services but were involved in teaching medical learners and/or doctors within their area of expertise.

Our data satisfactorily met the assumptions critical in conducting CFA. Skewness values ranged from −0.42 to −0.66, while kurtosis ranged from −0.09 to −0.59. Based on Finney and DiStefano [40], kurtosis and skewness not exceeding 7 and 2 respectively are considered normally distributed. There were no multivariate outliers. There were no missing data in the dataset. The descriptive statistics including the mean, standard deviation and Cronbach’s alpha were in the expected direction (see Appendix Tables 2 to 4).

**Table 1.** Fit indices of the WHO-5 for all participants (*N* = 435)

	*χ*^2^	*df*	χ^2^/*df*	*p*	CFI	NFI	IFI	TLI	RMSEA (90% CI)
WHO-5	18.425	4	3.09	0.026	0.99	0.99	0.99	0.98	0.08 (0.05, 0.14)

CFI = comparative fit index; NFI = Bentler-Bonett normed fit index; IFI = incremental fit index; TLI = Tucker-Lewis index; and RMSEA = root mean square error of approximation.

**Table 2.** Fit indices of the WHO-5 among educators with a medicine background (*n* = 333)

	*χ*^2^	*df*	χ^2^/*df*	*p*	CFI	NFI	IFI	TLI	RMSEA (90% CI)
WHO-5	8.83	3	2.94	0.032	0.99	0.99	0.99	0.98	0.07 (0.05, 0.14)

Note: CFI = comparative fit index; NFI = Bentler-Bonett normed fit index; IFI = incremental fit index; TLI = Tucker-Lewis index; and RMSEA = root mean square error of approximation.

**Table 3.** Differences in WHO-5 scores across age groups

Age Groups	Mean (*SD*)	*F*	*p*	*Scheffé Post-hoc comparison*
Group 1: 20–39 (*n* = 94)	56.51 (20.20)	8.04	<0.001	1 < 4 2 < 4
Group 2: 40–49 (*n* = 144)	55.86 (21.10)			
Group 3: 50–59 (*n* = 106)	62.79 (18.52)			
Group 4: 60 and above (*n* = 70)	68.84 (21.22)			

*SD* = standard deviation.

**Table 4.** Gender differences of the WHO-5

Instrument	Male (*n* = 280)	Female (*n* = 136)		
	Mean (*SD*)	Mean (*SD*)	*t* (414)	*p*
WHO-5	60.67 (21.19)	58.41 (19.85)	1.04	0.30

*SD* = standard deviation; *p* = empirical-level of two-tailed testing; *t* (414) = *t*-value with 414 degrees of freedom.

### Within-network construct validity

**Figure 1.**
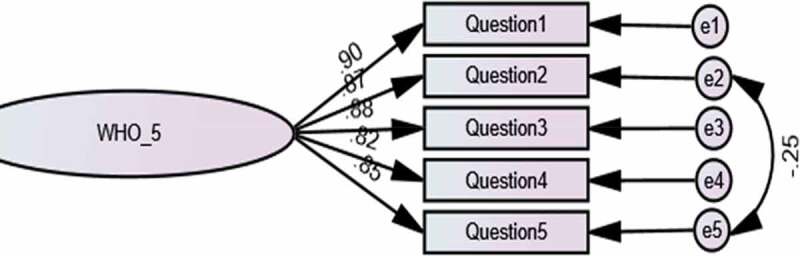
Confirmatory factor analysis of one-factor WHO-5 model.

Using the maximum likelihood estimation approach of CFA, we assessed the goodness-of-fit of the WHO-5 model to the participants’ data (Table 1, Figure 1). Model fit was checked following the recommended cut-off values as described in the data analysis section. The results of CFA were acceptable: CFI = 0.99, NFI = 0.99, IFI = 0.99 and RMSEA = 0.08 (CI 90% = 0.05 − 0.14).

We also performed a separate analysis for 333 participants with medicine as their professional background. The CFA results of this sub-group were similar to that of the entire sample with acceptable goodness-of-fit: CFI = 0.99, NFI = 0.99, IFI = 0.99 and RMSEA = 0.07 (CI 90% = 0.05 − 0.14) (Table 2). We were unable to conduct CFA separately on data from participants with other healthcare professional backgrounds due to the small numbers involved (*n* = 84).

To better understand the performance of the scale, we performed comparison of means. In particular, we examined whether the WHO-5 was able to differentiate between participants’ scores when grouped according to gender, age, and professional background. A one-way analysis of variance showed significant effect of age on well-being, *F*(4,415) = 8.04, *p* = <0.001 (Table 3). Post hoc analyses using the Scheffé post hoc criterion for significance indicated that Group 4ʹs (60 years old and above) well-being was significantly higher than Group 1 (20–39 years old) and Group 2 (40–49 years old). There was no significant gender-related difference in well-being between male (*M* = 60.67, *SD *= 21.19) and female (*M* = 58.41, *SD *= 19.85) medical educators; *t*(414) = 1.04, *p* = 0.30 (Table 4). There was a significant difference when participants were grouped by professional background, with the well-being of those in medicine (*M* = 61.23, *SD *= 19.61) being significantly higher than other healthcare professionals; *t*(415) = 2.84, *p* = <0.01 (Table 5).

**Table 5.** Differences in the WHO-5 by professional background

Instrument	Medicine (*n* = 333)	Other Healthcare Professionals (*n* = 84)		
	Mean (*SD*)	Mean (*SD*)	*t* (415)	*p*
WHO-5	61.23 (19.61)	54.10 (23.92)	2.84	<0.01

Other Healthcare Professionals include Biomedical Sciences, Nursing and Pharmacy; *p* = empirical-level of two-tailed testing; *t* (415) = *t*-value with 415 degrees of freedom.

### Between-network construct validity

To understand the between-network construct validity of the WHO-5, we used resilience as measured by CD-RISC2 as a predictor of well-being. Regression analysis revealed that resilience positively predicted well-being (*R*^2^ = 0.20, *F*(1,433) = 110.98, *p = *<0.001).

## Discussion

This study aims to offer preliminary evidence regarding the psychometric validity of the WHO-5 among a sample of 435 HK medical educators. The WHO-5 has often been used in Western settings but analyses of its applicability in Chinese-dominated cultures remain limited. Results in general point to the validity of the WHO-5, indicating that it is psychometrically sound and therefore, a valid instrument to use among the cohort of HK medical educators examined in this study.

The results of CFA performed to examine the within-network validity of the WHO-5 confirmed that it has one latent construct underpinned by five manifest variables. Its unidimensional structure has been confirmed using our current study’s data sample. The fit indices for this model were acceptable. The Cronbach’s alpha reliability for the WHO-5 was high and within the range reported in the literature [14]. In addition, the GFI, IFI, TLI and CFI were all above 0.90 and the RMSEA was adequate, suggesting acceptability of the fit indices.

To shed further light into the inherent psychometric ability of the WHO-5, we compared participants’ WHO-5 score by age group, gender and professional background. In terms of age, our results suggest educators 60 years old and above have significantly higher well-being than those in the younger age groups (i.e., 20–39 and 40–49 years old). This finding aligns with Nørøxe and colleagues’ study where older Danish general practitioners were noted to have higher well-being than their younger counterparts [17]. Similarly, Schindler and colleagues found that older academic physicians and basic medical science educators at four U.S. medical schools were less impacted by various stressors although other instruments were used in their research such as the Center for Epidemiology Study Depression Scale and the Rand Anxiety Scale [41]. Presumably, these educators had weathered significant changes and challenges throughout their careers in academia. In contrast, among frontline physicians and nurses caring for COVID-19 patients in Oman, no difference in well-being by age was noted [18] and in German emergency medical service professionals, older age was associated with poorer well-being [16].

In terms of gender, we did not find a difference between males and females in our current data. Our results are consistent with earlier studies where no significant gender differences in well-being were noted among Canadian resident physicians [19], American medical students, interview candidates for residency, and internal medicine residents plus faculty [20], as well as emergency department staff during the first COVID-19 wave in the Netherlands [21]. However, among German paramedics and emergency physicians [16] as well as the general working population, females were found to have poorer well-being [22,23].

In terms of professional background, our results demonstrated that medical doctors had significantly higher well-being than other healthcare professionals. This is in line with the Dutch study where emergency department doctors were found to have a significantly higher mean WHO-5 score compared to nurses, nursing assistants and administrative staff during the peak of the first COVID-19 wave [21]. However, we also acknowledge that there may be some sampling biases in our data set as the number of medical doctors was significantly higher than the combined number of other healthcare professionals (e.g., nurses, pharmacists). Furthermore, the relatively small number of other health professionals may have impacted the outcomes of the analysis.

To establish the between-network construct validity of the WHO-5, we clarified its relationship with other theoretically relevant variables. Theoretically, one of the areas previously explored is the nomological network of well-being where resilience is one of the antecedents [42]. Our results supported this relationship with resilience positively predicting well-being measured by the WHO-5. This finding is also congruent with previous studies reporting a link between the two variables [15,28,29]. In sum, our results serve as strong evidence that the validity of the WHO-5 can be extended to assess well-being among our cohort of HK medical educators.

Our study has several limitations to be considered. *First*, our findings may not be generalisable to healthcare professionals, academics or researchers who are not involved in medical education. *Second*, convenience sampling may have resulted in self-selection and sampling bias. *Third*, our results may be less applicable in other non-Asian cultures or settings although participants had varied ethnic backgrounds. *Fourth*, given that our data collection took place during the fourth wave of the COVID-19 pandemic in HK, we do not have the longitudinal data to support the stability of medical educators’ well-being after this wave of the pandemic. *Finally*, our study participants were from one of the two government-funded universities in HK with a medical school. The sociodemographic backgrounds of their academic staff, as well as institutional financial support and availability of resources appear to be similar [43,44]. Additionally, our sample size was greater than the minimum calculated by power analysis [45]. However, to enhance the generalisability of our findings, we invite other researchers to replicate our study so that medical educators from both HK medical schools are represented.

We also acknowledge that various important research areas of theoretical value are not covered in this study. Therefore, future research directions to consider include understanding the nomological network of the WHO-5. The ability of the WHO-5 to predict outcomes in various contexts would be another important line of research. Additionally, longitudinal research designs would be useful in following the trajectory of medical educators’ well-being during and after the COVID-19 outbreak. Lastly, examining the utility of the WHO-5 in practice to monitor the well-being of medical educators over time or in response to interventions and programmes to promote well-being would be valuable.

As a final note, we hope that the completion of this validation study becomes a springboard by which HK medical educators are represented in the intellectual discussion of well-being in the literature. With the growing interest in cross-country research into well-being, the challenge to produce psychometrically and psychologically sound scales is important. Hopefully, our initial work will catalyse opportunities to study HK medical educators’ well-being in depth to support them to perform even better in their teaching environments.

## Appendix

**Table 1.** Characteristics of participants

Participant characteristics (N = 435)	
**Age, no. (%)** ^a^	
20–29	8 (1.9)
30–39	86 (20.5)
40–49	144 (34.3)
50–59	106 (25.2)
≥ 60	70 (16.7)
Prefer not to say	6 (1.4)
**Gender, no. (%)** ^a^	
Male	280 (66.7)
Female	136 (32.4)
Prefer not to say	4 (1.0)
**Ethnicity, no. (%)** ^a^	
Chinese	394 (93.8)
Caucasian	9 (2.1)
Mixed/Multiple	4 (1.0)
Others	7 (1.7)
Prefer not to say	6 (1.4)
**Professional Background, no. (%)** ^a^	
Biomedical Sciences	31 (7.4)
Medicine	333 (79.9)
Nursing	30 (7.2)
Pharmacy	6 (1.4)
Other	17 (4.1)
**Highest Professional Qualification, no. (%)** ^a^	
Bachelor’s degree	29 (6.9)
Specialist qualification	186 (44.3)
Master’s degree	67 (16.0)
Doctorate	132 (31.4)
Other	6 (1.4)
**Years Worked as health professions educator, no. (%)** ^a^	
0–4	50 (11.9)
5–9	72 (17.1)
10–14	74 (17.6)
15–19	47 (11.2)
≥ 20	177 (42.1)
**Employment status, no. (%)** ^a^	
Full-time	255 (60.7)
Part-time	14 (3.3)
Honorary	148 (35.2)
Temporary	1 (0.2)
Other	2 (0.5)

^a^Numbers do not add up to 435 due to missing data; percentage is calculated based on the number of complete data.

## Table 2.

**Table 2.** Basic statistics of the scales

Scales	Range	Mean (*SD*)	Cronbach’s alpha
**WHO-5**	0–100	59.6 (20.9)	0.93
**CD-RISC2**	2–8	5.8 (1.3)	0.67
WHO-5 = 5-item World Health Organization Well-being Index; CD-RISC2 = 2-item Connor-Davidson Resilience Scale; *SD* = standard deviation.			

## Table 3.

**Table 3.** Internal consistency of WHO-5: Cronbach’s alpha of WHO-5, Corrected item-total correlation, and Cronbach’s alpha if the item is deleted from the scale

Item	Cronbach’s alpha	Corrected item-total correlation	Cronbach’s alpha if item deleted
WHO-5 (all 5 items)	0.93	-	-
1. I have felt cheerful and in good spirits.		0.86	0.91
2. I have felt calm and relaxed.		0.82	0.92
3. I have felt active and vigorous.		0.85	0.91
4. I woke up feeling fresh and rested.		0.79	0.93
5. My daily life has been filled with things that interest me.		0.80	0.92

Corrected item-total correlation is the correlation between each item and a scale score that excludes that item. Since all values are greater than 0.4, none of the items needs to be removed. Cronbach’s alpha if item is deleted is the value of overall alpha of each domain if that item is not included in the calculations. None of the items here would substantially affect reliability if they were deleted.

## Table 4.

**Table 4.** Inter-item correlation of the five items of the WHO-5

Item	1	2	3	4	5
1. I have felt cheerful and in good spirits.	-	0.78	0.79	0.70	0.75
2. I have felt calm and relaxed.		-	0.75	0.73	0.67
3. I have felt active and vigorous.			-	0.71	0.76
4. I woke up feeling fresh and rested.				-	0.71
5. My daily life has been filled with things that interest me.					-

Note: All values are significant (*p* < 0.001).
